# Identification and the potential involvement of miRNAs in the regulation of artemisinin biosynthesis in *A. annua*

**DOI:** 10.1038/s41598-020-69707-3

**Published:** 2020-08-12

**Authors:** Shazia Khan, Athar Ali, Monica Saifi, Parul Saxena, Seema Ahlawat, Malik Zainul Abdin

**Affiliations:** Department of Biotechnology, School of Chemical and Life Sciences, Centre for Transgenic Plant Development, Jamia Hamdard, New Delhi, 110062 India

**Keywords:** Computational biology and bioinformatics, Genetics, Molecular biology, Plant sciences

## Abstract

Micro RNAs (miRNAs) play crucial regulatory roles in multiple biological processes. Recently they have garnered the attention for their strong influence on the secondary metabolite production in plants. Their role in the regulation of artemisinin (ART) biosynthesis is, however, not fully elucidated. ART is a potent anti-malarial compound recommended by WHO for the treatment of drug-resistant malaria. It is produced by *Artemisia annua* (*A. annua*)*.* The lower *in planta* content of ART necessitates a deep understanding of regulatory mechanisms involved in the biosynthesis of this metabolite. In this study, using modern high throughput small RNA-sequencing by Illumina Nextseq 500 platform for identification and stem-loop RT PCR for validation, miRNAs were identified in the leaf sample of *A. annua* plant. Here, we report a total of 121 miRNAs from *A. annua* that target several important genes and transcription factors involved in the biosynthesis of ART. This study revealed the presence of some important conserved miRNA families, miR396, miR319, miR399, miR858, miR5083 and miR6111 not identified so far in *A. annua.* The expression patterns and correlation between miRNAs and their corresponding targets at different developmental stages of the plant using real-time PCR indicate that they may influence ART accumulation. These findings thus, open new possibilities for the rational engineering of the secondary metabolite pathways in general and ART biosynthesis in particular.

## Introduction

*Artemisia annua* (*A. annua*), an important medicinal herb is the only natural source of artemisinin (ART). ART, a sesquiterpene lactone is the most reliable and widely used drug to quell Malaria, which is one of the most devastating diseases and a leading cause of deaths worldwide. According to WHO, there were a staggering 228 million cases and 405,000 deaths reported in 2018 alone, despite the disease being preventable as well as curable^1^. WHO has recommended the use of ART only in combination of other drugs, to slow down the development of resistance in *Plasmodium* sps. against it and to maintain its efficacy. The combination treatment is widely known as artemisinin-based combination therapies (ACTs). In wild type *A. annua,* ART is present in the aerial parts, in extremely low quantities (0.02–1.07%)^2^. Numerous efforts were made to generate ART synthetically or semi-synthetically, but does not meet industrial practices due to high production cost. The insufficient supply of ART produced by plants or synthetic methods, resulted in the lack of ACTs available to the patients and causes approximately half a million mortalities every year^3^. Despite various efforts to produce semi-synthetic ART at low cost, the natural source of ART remains the main provider. Thus, researchers are investigating effective strategies to enhance *in planta* content of ART. The Ian Grahm lab and Bill and Malinda gates foundation invested huge fund in production of high ART yielding hybrids and succeeded in development of HYB8001R that could produce 1.4% ART of dry weight^4^. Reconstitution of the pathway and increasing the area under cultivation could be a promising solution to increase ART production. Ding et al., 2020 suggested the potential fields suited best for the cultivation of *A. annua* plant worldwide. They found that contrary to the higher demands in African region the potential fields are minimal. The best suited land resources were predicted to be distributed around the south eastern coast of Brazil and Australia^5^. Thus, the strong international cooperation is needed to resolve this spatial mismatch.

The reconstitution of the pathway, requires the complete elucidation of the biosynthetic pathway genes and regulatory elements. In plants, the secondary metabolites biosynthesis and accumulation is highly regulated. ART biosynthesis has been extensively studied and it was found that two metabolic pathways; cytoplasmic mevalonic acid (MVA) and plastidial methylerythritol phosphate (MEP) are involved in providing the basic carbon precursors of isoprene units for the biosynthesis of ART. To date, genes encoding enzymes of MVA and MEP pathways such as HMGR, IDI, DXS, DXR, HDR and FPS as well as genes for all five downstream enzymes of ART biosynthesis pathway namely ADS, CYP71AV1/CPR, ADH1, ALDH1, and DBR2 are over-expressed in *A. annua* plants^6–13^. Shen et al. (2018) showed up to 3.2% enhancement in the production of ART in *A. annua* transgenic lines that were simultaneously overexpressing the FPS, HMGR and DBR2 genes^14^. Role of TF in the regulation of complex pathways is well known and cannot be neglected, when a lot of reports are advocating their regulatory role in secondary metabolite biosynthesis. Consequently, TFs were cloned and their effect on ART content was studied. It was shown that AaMYB1 significantly enhance the transcription of key genes of the ART biosynthetic pathway, especially ADS and CYP71AV1, that resulted in an increased ART content in transgenic plants^15^. Similar results were obtained when other TFs such as AaERF1, AabZIP, bHLH, AaWRKY, AaMYC2, AaGSW1, AaORA and AaTAR1 were overexpressed^16–19^. Due to the fine tuning of pathways and probably due to the presence of other key players, these efforts were however, unable to effectively boost up the entire metabolic flux towards ART biosynthesis. In a study, it was however, shown that the overexpression or loss of miR163 alters entire secondary metabolite profile in mir163 mutant of *Arabidopsis*^20^. Also, another study revealed that miR156-trageted SPL9 directly binds terpene synthase promoter and regulate β-caryophyllene synthesis in *Arabidopsis*^21^. Thus, it seems reasonable to assume that miRNAs can also be used as effective regulators for modulating ART biosynthesis based on their targets in ART biosynthetic pathway. Here, we are reporting, miRNAs from *A. annua* and their possible involvement in ART biosynthesis.

## Results

### Sequencing of small RNAs and data analysis

**Figure 1:**
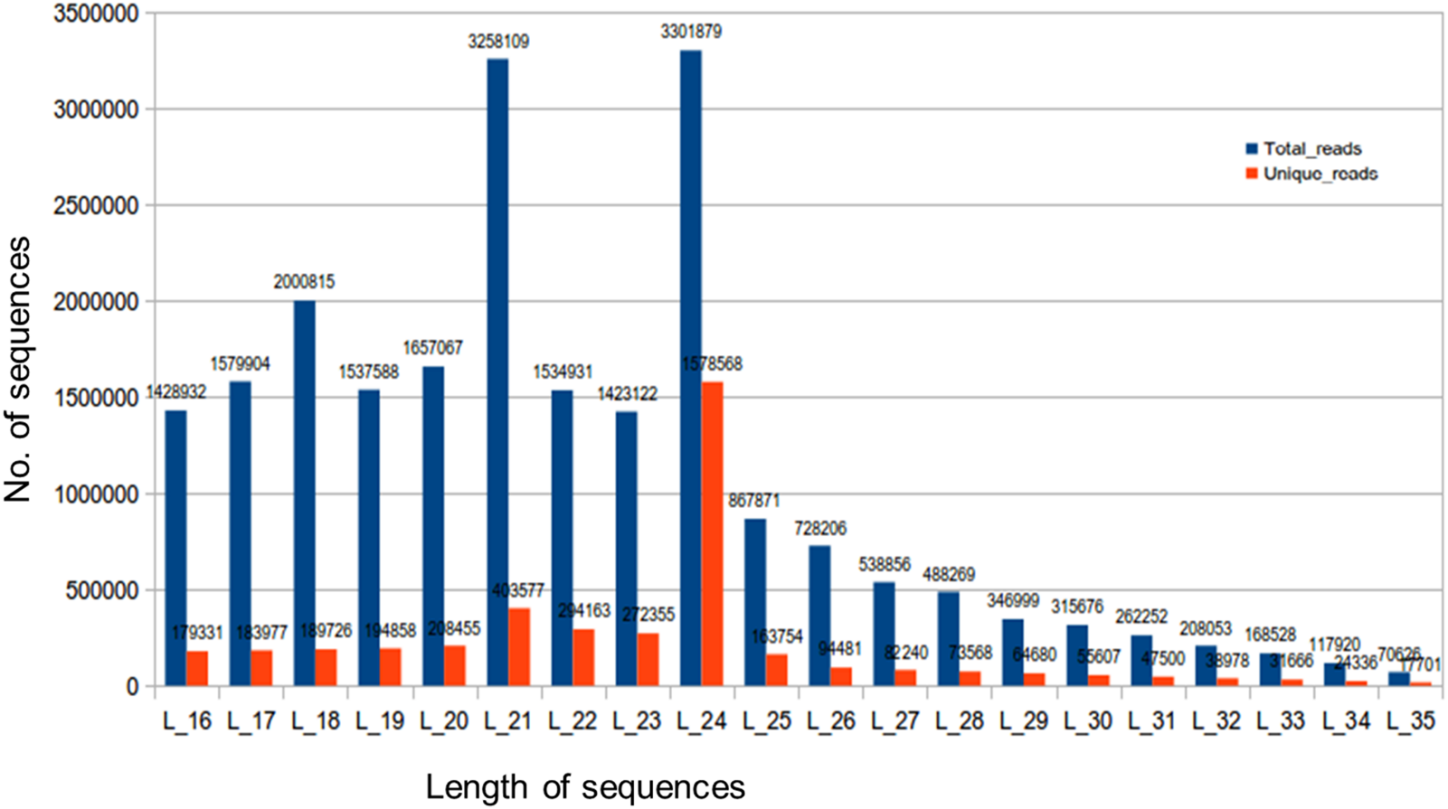
Length distribution graph of small RNA sequences in leaf sample library of *A. annua.*

A high-quality RNA (RIN number 8.4), isolated from the leaves of *A. annua* at pre-flowering stage was subjected to next generation sequencing (NGS), thereby resulted in generation of 36,392,552 raw reads. As a result of further analysis, i.e. selection of size, trimming of adapters and low-quality reads, 34,741,778 clean reads (unique reads 5,228,750) were remained. Further removing the other non-coding RNA sequences such as snRNA, siRNA, tRNA, rRNA, snoRNAs resulted in 21,835,603 clean reads (unique reads 4,199,521). Among the clean reads the maximum reads were of 24nt followed by 21nt and then 20, 22 and 23 nt reads. The length distribution graph is given in Fig. 1. The 23 and 24nt sequences possess 5′-adenosine at the first position, generally considered as endogenous siRNAs ^22^. These are one of the most abundant classes of small RNAs in plants, while most plant miRNAs tend to be 21 and 22nt in length ^23^.

### Identification of *A. annua* conserved and novel miRNAs

The clean reads obtained were subsequently analysed to predict conserved and novel miRNAs in *A. annua*. As a result, 121 miRNAs were identified, 80 of which were conserved across plant species whereas, 41 candidates were predicted as *A. annua* specific novel miRNAs (supplementary file S1). The conserved miRNAs belong to 23 different families of miRNAs. Varying level of miRNA expression was found in the library. Out of all, the maximum expression was observed for miRNA396f, followed by miRNA159a with 261,298 and 73,683 reads, respectively. The ten most abundant miRNAs in the sample are shown in Table 1.

**Table 1:** Ten most abundant conserved miRNA families in *A. annua* leaf sample library.

S. No	MicroRNA	Abundance (reads per million)
1	miR396f	261,298
2	miR159a	73,683
3	miR166a-3p	47,883
4	miR166m	19,512
5	miR166n	19,240
6	miR396g-5p	11,558
7	miR396b-5p	8,444
8	miR166g-3p	6,714
9	miR162a-3p	5,741
10	miR159a.1	4,930

A total of 248 sequences of 18–22 nt were also identified that were either 1-2nt shorter or longer than miRNA in miRbase-21 (supplementary file S1). In our data, the highest number of miRNAs belongs to miR166 and miR396 families having 16 members and 12 members respectively. While, families miR170, miR171, miR393, miR394, miR858, miR5083, miR5368, and miR6111 were found to have only one member. Number of members in each family are summarized in Table 2.

**Table 2:** No. of members in conserved miRNA families in *A.annua.*

S. No.	miRNA family	No. of members
1	miR166	16
2	miR396	12
3	miR159	9
4	miR162	5
5	miR156	4
6	miR167	4
7	miR319	4
8	miR398	4
9	miR160	2
10	miR164	2
11	miR165	2
12	miR168	2
13	miR172	2
14	miR399	2
15	miR403	2
16	miR170	1
17	miR171	1
18	miR393	1
19	miR394	1
20	miR858	1
21	miR5083	1
22	miR5368	1
23	miR6111	1

Furthermore, a total of 41 sequences were successfully identified as novel miRNA candidates in *A. annua*. These sequences have a length ranging from 18 to 24 bp, but the maximum among them were 20–22 bp long. The minimum fold energy (MFE) observed was between − 18.3 kcal-mol^−1^ to − 84.4 kcal-mol^−1^. This showed good agreement with previous reports on novel miRNA predictions in other species (MFE of *Catharanthus roseus*, − 43.2 kcal-mol^−1^; Opium poppy (*Papaver somniferum*), − 34.09 kcal-mol^−1^; pigeonpea (*Cajanus cajan*), − 34.8 kcal-mol^1^; *Arabidopsisthaliana*, rice (*Oryza sativa*), soybean (*Glycine max*), *Medicago truncatula, Saccharum officinarum*, sorghum (*Sorghum bicolor*) and maize (*Zea mays*) from − 40 to 100 kcal-mol^−1^)^24–27^. The minimum fold energy index (MFEI) is an important criterion to characterize miRNAs. When the MFEI is more than 0.85, the sequence is most likely to be miRNA. It was observed that the MFEIs of stem loop structures of the miRNAs were ranged from 0.87 to 2.1. All the information along with precursor sequences is given in supplementary file S1.

### Validation of conserved and novel miRNAs

**Figure 2:**
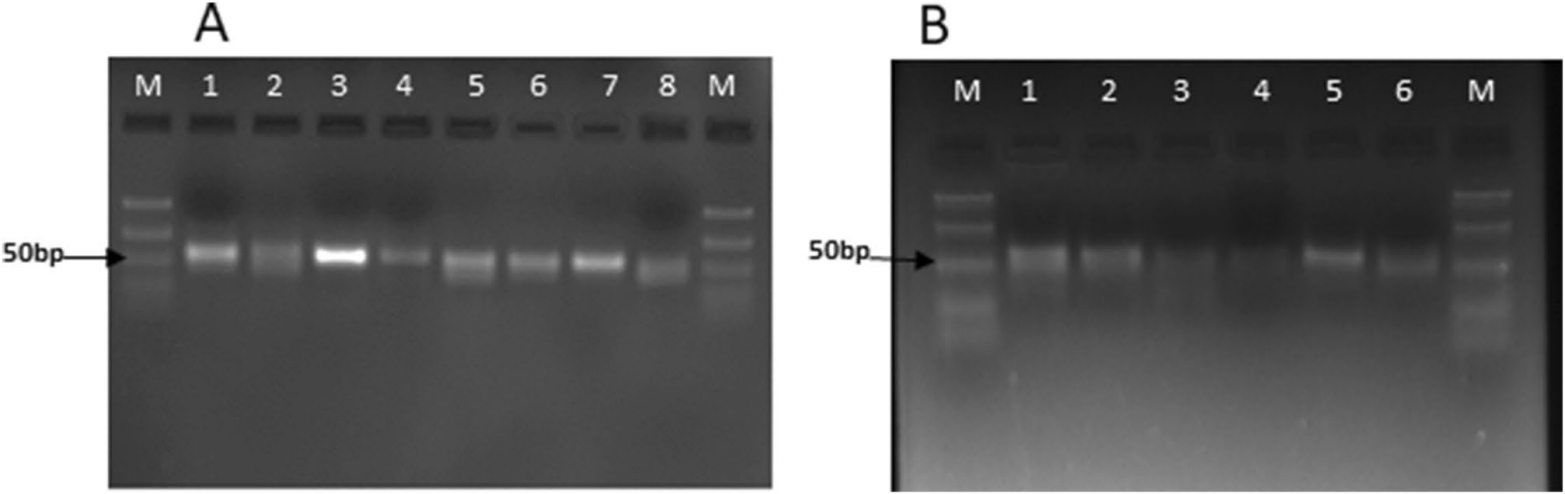
Agarose gel based detection of miRNAs **(A)** conserved miRNAs M:50 bp ladder, 1: miR5083, 2: miR858b, 3: miR159a, 4: miR396, 5: miR172a-3p, 6: miR166i, 7: miR166g, 8: miR162 **(B)** novel miRNAs M: 50 bp ladder, 1:miRn-7, 2: miRn-10, 3: miRn-11, 4: miRn-18, 5:miRn-29, 6: miRn-26.

The miRNAs obtained by NGS were validated using stem-loop PCR according to published protocol^28^. Of the 15 selected miRNAs (8 conserved and 7 novel miRNAs), we confirmed the expression of 14 miRNAs (Fig. 2).

To further strengthen our results, all these miRNAs were also checked at different developmental stages of the plant. All these miRNAs were successfully amplified in all the stages. Thus, the results obtained in this experiment and the data obtained by NGS present enough support for the existence of these miRNAs in *A. annua*.

### Target prediction

As a result of target prediction, 54 unique targets of 52 conserved miRNAs, and more than 60 targets of 30 novel miRNAs were successfully identified. Results are given in supplementary file S1. Target interpretation was mainly focused on their putative roles in ART biosynthesis. Multiple miRNAs were found to target single mRNA. For instance, three families of miRNAs, namely miR159, miR166, miR172, and four novel miRNAs namely miRn-8, miRn-20, miRn-36 and miRn-37 putatively targets cytochrome p450 reductase (CPR) mRNA. CPR is a redox partner of CYP71AV1, an important enzyme in ART biosynthesis. Another important miRNA family is miR396 family. The three members of this family, viz. miR396, miR396f and miR396g-5p targets DXR1, a rate limiting enzyme of plastidial MEP pathway. Similarly, other enzymes of MVA and MEP pathways such as HMGR, IDI, DXS, HDR and FPP are targeted by several miRNAs. The details are given in supplementary file S1. These enzymes play an important role in producing basic isoprene units for production of all terpenoids. It was observed that miR858 putatively target transcript for the enzyme ADS. Other significant miRNAs were miRn-11, miRn-17, miRn-18, that targets CYP71AV1 mRNA. Both of the enzymes are key players in ART biosynthesis and thus, these miRNAs could play crucial roles in the ART biosynthesis regulation. In addition to above mentioned miRNAs, we have identified miRNAs that targets TFs. We observed that miR858 targets transcription factor family MYB. In *A. annua*, MYB1 is involved in ART biosynthesis regulation^15^. It was found that in *Arabidopsis,* miRNA858 targets several members of MYB family that regulate the synthesis of flavonoids^29^. Consistent with these reports, miR858 in *A. annua* targets various members of MYB. In addition, miR858 was also predicted to target ADS transcript. ADS is one of the key enzymes in ART biosynthesis regulation. Another important TF, NAC1 is a putative target of miRNA families 403 and 5,083. In literature, NAC1 is reported to regulate two key genes ADS and CYP71AV1 of ART biosynthesis pathway. WRKY1 the third TF putatively targeted by miR166g is also reported to drag ART biosynthesis pathway in positive direction and help in accumulation of ART by regulating pathway genes. Similarly, MYC2 is the putative target of miRn-26 and miRn-41. Apart from targeting transcripts of gene, few miRNAs targets promoter region of the genes. This includes miR159, miR172a-3p, miR166i, miRn-7, miRn-10, miRn-24, and miRn-29. Of which miR166i and miRn-7 target DBR2 gene promoter; miR159, miR172a-3p and miRn-10 target ERF1 gene promoter while miRn-24 and miRn-29 target promoters of WRKY1 and ADS genes, respectively.

**Figure 3:**
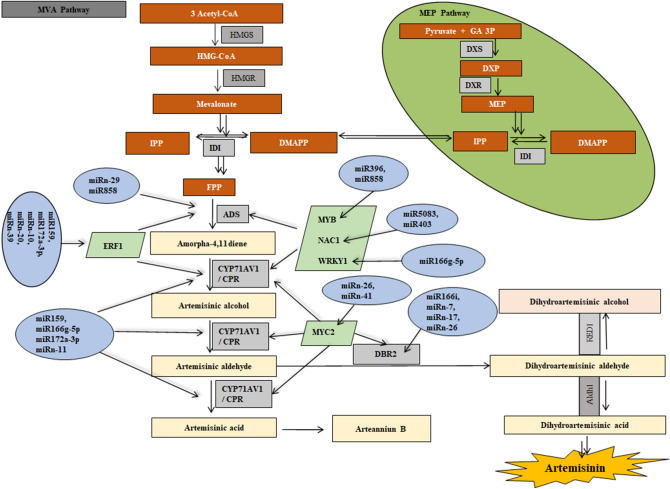
ART biosynthesis and putative involvement of miRNAs. The MVA pathway and MEP pathway products are shown in orange boxes. *HMG-CoA* hydroxymethylglutaryl CoA, *IPP* isopentenyl diphosphate, *DMAPP* dimethylallyl diphosphate, *FPP* farnesyl diphosphate, *DXP* 1-deoxy-d-xylulose 5-phosphate, *MEP* methyl erythritol phosphate, enzymes are indicated in grey boxes, *HMGS* HMG-CoA synthase, *HMGR* HMG-CoA reductase, *IDI* IPP isomerase, *DXS* 1-deoxy-d-xylulose 5-phosphate synthase, *DXR* DXP reductoisomerase, *ADS* armorpha-4, 11-diene synthase, *CYP71AV1* cytochrome P450 mono-oxygenase, *CPR* cytochrome P450 reductase, *DBR2* artemisinic aldehyde delta-11(13)-double bond reductase, *ALDH1* aldehyde dehydrogenase, *RED1* dihydroartemisinic aldehyde reductase, transcription factors are indicated in green boxes and miRNAs are indicated in blue circles.

Additionally, we have found that the mRNAs of β-amyrin synthase, β-farnesene synthase, and linalool synthase, which are involved in the formation of other terpene molecules are targeted by some conserved and *A. annua* specific novel miRNAs listed in supplementary file S1. Many researchers showed that blocking the pathways involved in biosynthesis of other terpene molecules, redirect the carbon flux towards ART biosynthesis in *A. annua*. Some important miRNAs involve in ART biosynthesis were given in Fig. 3. The detailed target prediction data is available in supplementary file S1.

### Differential expression and correlation of miRNAs and their putative targets

**Figure 4:**
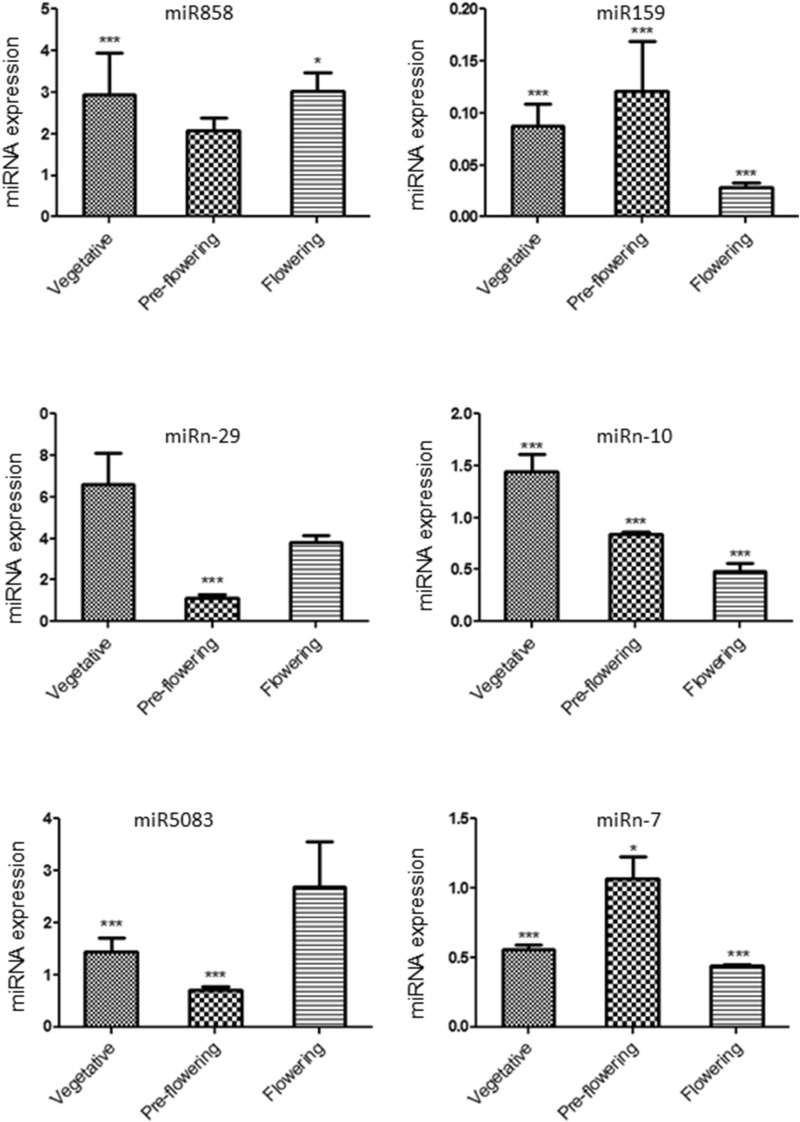
Relative expression of selected miRNAs levels quantified by real-time PCR at vegetative, pre-flowering and flowering stages of the *A. annua* plant. The expression levels of miRNAs were normalized to the expression level of U6 RNA. Relative expression was calculated using 2^^− ΔCt^ equation, where Ct = (Ct_miRNA_ − Ct _U6_). ANOVA followed by Dunnett’s test was applied to all miRNAs compared to U6. The error bar shows the standard error. *P < 0.05, ***P < 0.001.

**Figure 5:**
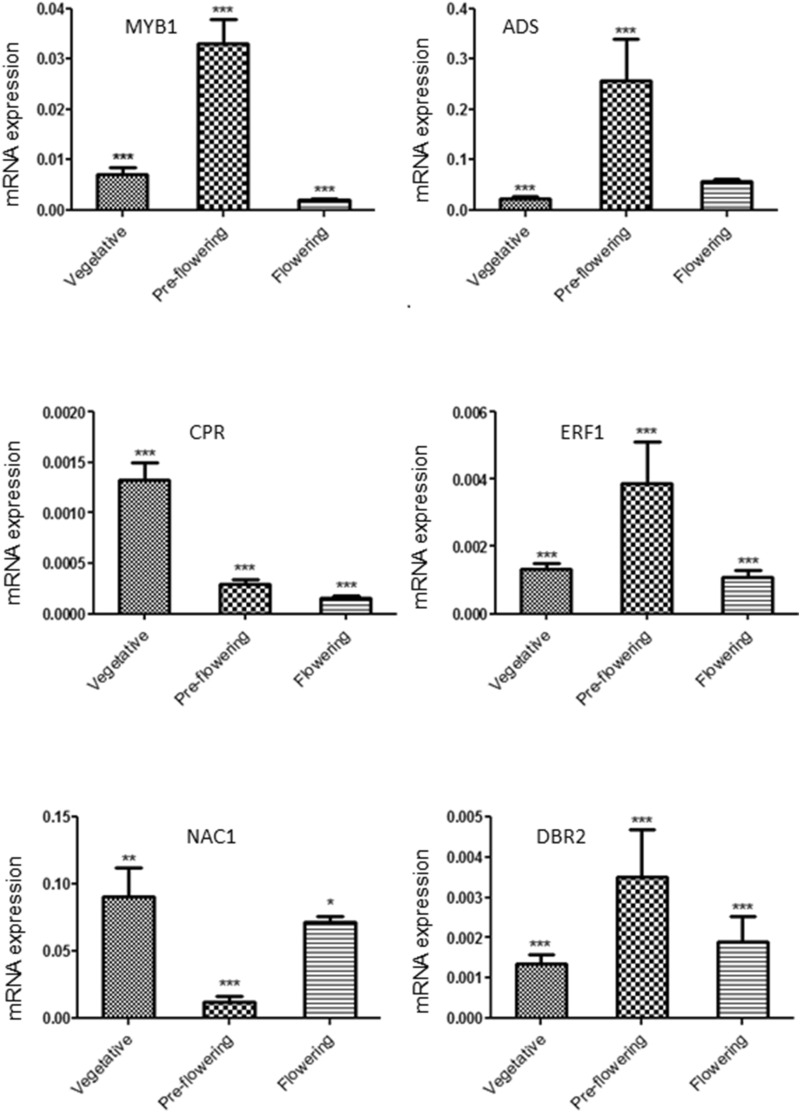
Relative expression of selected mRNAs levels at vegetative, pre-flowering and flowering stages of the *A. annua* plant. The expression levels of mRNAs were normalized to the expression level of β-actin. Relative expression was calculated using 2^^− ΔCt^ equation, where Ct = (Ct_gene_ − Ct _β-actin_). ANOVA followed by Dunnett’s test was applied to all mRNAs compared to β-actin. The error bar shows the standard error. *P < 0.05, **P < 0.01, ***P < 0.001.

The expressions of six miRNAs, viz. miR858, miR5083, miR159, miRn-7, miRn-10, miRn-29 and their putative target genes evaluated at different developmental stages viz*.,* vegetative, pre-flowering and flowering stages of the plant using qRT-PCR are shown in Figs. 4 and 5. Results showed that miR858 was negatively correlated with its targets MYB1 and ADS (Pearson coefficient − 0.52, − 0.50). Similarly, miR159 was negatively correlated with its target CPR (Pearson coefficient − 0.68). Interestingly, the expression of miR5083 was positively correlated with its target NAC1 (Pearson coefficient + 0.4). Of the four miRNAs viz. miR159, miRn-7, miRn-10, and miRn-29 that target promoter regions of ERF1, DBR2 and ADS genes, the expression levels of miRn-7 had a positive correlation with its target gene DBR2 expression (Pearson coefficient + 0.39). The expressions of miRn-10, miR159 and miRn-29 were not found to be correlated with the expression of their target genes.

## Discussion

Micro RNAs are small non coding RNAs that exert extensive impact on the regulation of gene expression in both plants and animals. In recent years, they have garnered attention due to their involvement in a number of complex biological processes including the secondary metabolite production in plants^30^. Their possible involvement in ART biosynthesis is however, yet to be elucidated. Previous three reports on miRNAs identification and target prediction in *A. annua* were based on computational identification and predicted only six, thirteen and eleven miRNAs, respectively that may represent only a fraction of its miRNAome^31–33^. Here, we sought to identify and validate miRNAs present in *A. annua* plant using NGS along with qPCR. Modern deep sequencing/NGS has many advantages over computational prediction specially in species that do not have full genome sequences. A total of 121 miRNAs were identified that include some important conserved miRNAs like miR396, miR319, miR399, miR858, miR5083 and miR6111, which were not identified so far in *A. annua*. It was recently shown that miR319 regulate TCP4 proteins, involved in suppression of trichome branching in *Arabidopsis thaliana*^34^. Trichomes are factories for synthesis and accumulation of most secondary metabolites including ART^35,36^. Besides, reports on miR858 family mediated regulation of secondary metabolites are accumulating in model as well as non-model plants^37,38^. Thus, identification of these new classes of miRNAs may provide new directions to unveil the regulatory mechanisms controlling secondary metabolites biosynthesis and accumulation, and for rational engineering of ART biosynthetic pathway. NGS also allowed us to predict 41 novel miRNAs, based on their hairpin structures. Expression of *A. annua* specific novel miRNAs were lower as compared to conserved miRNAs, which is often the case of species-specific miRNAs^22,39^. Validation of fifteen miRNAs by stem-loop RT-PCR at different developmental stages of the plant further strengthen our NGS data.

Interestingly, some of the miRNAs target multiple genes, for instance, miR396e had eight targets while others like miR159a, miR159b, miR159c and miR159f were found to target the mRNA of the same gene. These results exhibit the complex miRNA-target network in *A. annua*.

This study was focused on identification of miRNAs involved in the regulation of ART biosynthesis. Results showed that three families of conserved miRNAs, viz*.* miR159, miR172 and miR166 were targeting a *cytochrome P450 reductase* (*CPR*) gene. CPR, along with CYP71AV1 catalyzes three steps of ART biosynthesis, oxidizing amorpha 4,11 diene to artemisinic acid^40,41^. qPCR data suggested that higher expression of miR159 is negatively correlated with target CPR gene expression (Pearson coefficient -0.68) might play a role in the repression of *CPR* gene at the pre-flowering stage. Another significant miRNA, miR858 targets ADS mRNA. ADS is an important enzyme in ART biosynthesis regulation. Expression of miR858 at different developmental stages is negatively correlated with ADS gene expression (Pearson coefficient − 0.50). miR858 was also targeted MYB TF as discussed later in this section. Besides, we also identified the nine *A. annua* specific miRNAs targeting important genes in ART biosynthesis.

Some of the TFs and miRNAs, while independently regulating their targets, collaborate with each other to regulate gene expression. Literature showed the involvement of at least seven families of TFs in ART biosynthesis that include AP2/ERF, bZip, bHLH, NAC, WRKY, MYC and MYB families^15,16,42,43^. In our results, it was found that miR858 targets several MYB TFs. The qPCR results revealed that the miR858 accumulation is negatively correlated with that of MYB1 (Pearson coefficient − 0.52), when checked at different developmental stages of the plant. Hernández et al.^15^ predicted the binding sites of R2R3 type of AaMYB1 in ADS, CYP71AV1, DBR2 promoters. They showed that the content of ART doubles in transgenic lines when AaMYB1 was cloned and overexpressed. Evidences on regulation of MYBs by miR858 are accumulating in plants such as *Arabidopsis, Actinidia arguta* (kiwifruit), *Vitis vinifera* (grapes), and *Ipomoea batatas* (sweetpotato), where they play key roles in the synthesis of secondary metabolites like anthocyanin and flavonoids^37,38,44,45^. It was also found that miR858 is involved in fungal susceptibility, and inhibiting miR858 by miRNA mimic technique trigger fungal resistance in *Arbidopsis thaliana*^46^. We speculate that miR858 may play similar roles in *A. annua*. NAC1, another important TF involved in ART biosynthesis was found to be the target of two families of conserved miRNAs, miR403 and miR5083. Most of the plant miRNAs exert negative regulation on their target genes. There are findings where their expressions are however, positively correlated. For example, Kawashima et al.^47^ revealed that the expressions of miR395 and its target gene, sulphate transporter (SULTR2;1) were positively correlated in the roots of *Arabidopsis* plants. Thus, it was interesting to discover a positive correlation in the expression of miR5083 with its target NAC1 gene (Pearson coefficient + 0.4). Similar kind of correlations were also reported in other living systems, suggesting that this is not restricted only to plants^48^. miR166g targets AaWRKY1 that regulates ART biosynthesis by binding to the W box in the ADS promoter^49^. It was shown that transgenic lines overexpressing AaWRKY1 lead to accumulate 1.9-fold higher ART content^50^. Similarly, TF AaERF1 was the putative target of miR159, miR172a-3p, miRn-10, miRn-20, and miRn-39, while two miRNAs, miRn-26 and miRn-41 could bind MYC2. We speculate that it will be more rational to modulate the TFs that regulate the multiple genes of ART pathway, by targeting miRNAs than to overexpress individual gene to enhance ART content.

Target prediction has led us to identify few miRNAs that target promoter regions of the important genes involved in ART biosynthesis. One of such miRNAs is miRn-7 that targets DBR2 gene promoter region. Induction of DBR2 gene leads to higher accumulation of ART^10^. Similarly, miRNA159, miR172a-3p, miR166i, miRn-24 and miRn-29 target the promoter regions of ERF1, DBR2 WRKY1, and ADS genes. It was observed that miRNAs that target the promoters of genes, activate their expression at the transcriptional level. For instance, miR5658 directly activates AT3G25290 expression by targeting its promoter in *Arabidopsis thaliana*.^51^. Similarly, Place et al.^52^ reported that miR-373 induces the expression of two genes, cold-shock domain-containing protein C2 (CSDC2) and E-cadherin by binding the promoters in PC-3 prostate cancer cells^52^. We observed that the expression of miRn-7 is positively correlated with the expression of its target gene DBR2 (Pearson coefficient + 0.39) at different developmental stages of *A. annua* plant. We presume that it could play important regulatory role in ART biosynthesis. The expression of miRn-29, miRn-10 and miR159 were not correlated with the expression of their target genes (Pearson coefficient < 0.3).

Apart from miRNAs targeting ART biosynthesis, it was interesting to discover microRNAs that target genes involved in biosynthetic pathways of other secondary metabolites. These pathways interact with ART pathway while competing for the common precursors generated by MVA pathway. We found that one conserved miRNA, miR162 was putatively targeting chalcone synthase mRNA while five novel miRNAs namely, miRn-1, miRn-4, miRn-18, miRn-20, miRn-26 were targeting chrysanthemyl synthase, cinnamyl alcohol dehydrogenase, β-amyrin synthase, linalool synthase and β-farnesene synthase mRNAs, respectively. Researchers showed that by inhibiting the pathways competing for the common precursors lead to divert the carbon flux towards ART biosynthesis and resulted in accumulation of higher ART in transgenic *A. annua* plants^53,54^. Further studies would however, be required for assessing the impact of these miRNAs on secondary metabolite profile. Thus, this study provides a new insight into the possible miRNA-directed molecular processes that regulate ART biosynthesis in *A. annua*.

## Methods

To identify miRNAs in the wild type *A. annua*, ‘cim-arogya’ variety was grown in the herbal garden of Jamia Hamdard, New Delhi, India. The seeds of these plants were obtained from Ipca Laboratories Pvt Ltd, M.P., India. It was a high artemisinin producing variety and contains 0.9 to 1.1% of artemisinin content^55^. Plant leaf samples at pre flowering stage were collected for RNA isolation.

### RNA extraction

For RNA extraction, leaf samples were grounded with mortar and pestle in liquid nitrogen. RNA was isolated using TRIzol reagent (Invitrogen, USA) as per manufacturer’s instructions. Total RNA integrity was checked on 1% formaldehyde gel and concentration of RNA was determined using nanodrop electrophotometer (Thermo Fischer Scientific, USA) while the RNA quality was checked on the BioAnalyzer (Agilent, USA).

### Library preparation and sequencing

RNA sample were sent for high throughput sequencing to M/S Sandor Lifesciences Pvt Ltd, Hyderabad, India. Small RNA library was generated using Illumina NextSeq 500 platform. NEB adaptor having sequence ‘AGATCGGAAGAGCACACGTCT’ was added for sequencing. Redundant reads were counted and reads per million (RPM) values were calculated. Low quality reads were removed before processing data. Raw reads were then processed, and adapter sequences were trimmed using Cutadapt tool. Remaining reads were then searched against Rfam database to eliminate other non-coding RNAs like rRNA, tRNA, snRNA and snoRNAs.

### Identification of *A. annua* miRNAs

The clean reads thus obtained were used to predict conserved and novel miRNAs. For conserved miRNA prediction clean reads were aligned to miRBase-21 (https://www.mirbase.org/) allowing up to 2 mismatches. The unaligned sequences left were used to predict novel miRNAs. At the time of sequencing the sRNAs, the genome of *A. annua* was not available. Although, it has been assembled in 2018, but the unavailability of complete annotation limits its application as good reference genome. Thus, for novel miRNA prediction, unique sRNA sequences were mapped to the EST sequences of *A. annua* and transcriptome of *A. annua* submitted to the National Center for Biotechnology Information (NCBI) database (https://www.ncbi.nlm.nih.gov/)*.* Mature as well as stem-loop structures were predicted for novel miRNAs using mireap_0.2 software. The criteria used were as follows: (i) The putative mature miRNAs were required to have length between 18 and 24 bases and at least have reads per million (RPM) > 2; (ii) There were no more than five mismatches and four bulges between mature miRNA candidate and the opposite hairpin arm; and (iii) the upper limit of minimum fold energy (MFE) of miRNAs was set to − 18 kcal-mol^−1^. Hairpin loops of all novel miRNAs were constructed by mfold web server using RNA folding form.

### Target search of *A. annua* miRNAs

The functional annotation of miRNAs was obtained using psRNATarget^56^, one of the most reliable and widely used tool to predict the targets gene of the miRNAs in plants with default parameters. *A. annua* nucleotide sequences from NCBI database (https://www.ncbi.nlm.nih.gov/unigene) were taken as data set for predicting the targets.

### Stem-loop RT-PCR

Results obtained from the next generation sequencing (NGS) were validated using stemloop RT-PCR, one of the most commonly used and reliable method for determination of miRNAs. Fifteen miRNAs were selected for validation by stemloop RT-PCR according to the published protocol^28^. The basis of selection of miRNAs was their targets in the artemisinin biosynthesis pathway. Amplification was obtained using standard PCR. Stemloop RT primers were designed for each of the fifteen miRNAs for reverse transcription and cDNA was prepared using RevertAid cDNA synthesis kit (Thermo Fischer scientific, USA). Reverse transcription reaction mixture was kept in thermal cycler (Thermo fischer scientific) under following condition: 1 cycle of 16 °C for 30 min; 60 cycles of 30 °C for 30 s, 42 °C for 30 s, 50 °C for 1 s, and 1 cycle of 70 °C for 15 min as described by Gasic et al. ^28,57^.

### Relative expression profiling of miRNAs and their putative target genes in ART biosynthesis pathway

To analyse the possible association of miRNAs and their putative target genes in ART biosynthesis pathway, expression of six selected miRNAs as well as their target genes were analysed by RT-qPCR. The basis of selection of miRNAs was their putative targets in ART biosynthesis pathway as well as their specificity. The miRNAs having only one or two targets were selected for expression analysis.

The leaf tissues from different developmental stages were taken. The stages of growth were considered from the days after transplanting (DAT). Leaves were collected at vegetative (90 days from DAT), pre-flowering (180 days from DAT-budding stage), and flowering stages (200 days from DAT) for RNA isolation. Three biological replicates were taken from each stage. Primers for miRNAs were designed as described by Gassic et al., and the primers for the genes were designed using IDT Primer quest tool. Primer sequences used in the study are given in supplementary file S1. Total RNA was isolated using TRIzol reagent (Invitrogen, USA). 1 µg of high-quality RNA (RIN 0.8–0.95) was taken for reverse transcription reactions in 20 µl reaction mixture using RevertAid cDNA synthesis kit (Thermo Scientific, USA).

After RT reaction cDNAs were diluted 10 times. The reactions for qPCR were performed in Roche light cycler 480 (Roche) with SYBR Green PCR Master Mix (Roche) in 10 µl tubes on 96 well plates. All reactions were replicated three times. To normalize the data U6 and β-actin were taken as internal controls for miRNAs and genes respectively. To finally calculate the relative expression, 2^^− ΔCt^ equation was used, where Ct = (Ct_miRNA_ − Ct_U6_) for miRNAs and Ct = (Ct_gene_ − Ct_β-actin_) for genes. The specificity of amplification was checked using melt curve analysis; no primer dimers were observed.

### Statistical analysis

The significant differences of miRNAs and their target genes with their reference genes at different developmental stages of the plant were calculated. Analysis was performed using one-way ANOVA followed by Dunnett's post hoc test. The data are presented as means ± S.E. The correlation between each miRNA and its target mRNA was investigated using the Pearson correlation test. A perfect negative correlation was defined as r =  − 1 and a perfect positive correlation as r =  + 1. Correlation values between -0.3 to + 0.3 were taken as no correlation between miRNA and their targets^58^. Results were considered statistically significant, when *p* < 0.05. All statistical analysis were performed using the GraphPad Prism 5.00,GraphPad Software, Inc., La Jolla, CA, USA^59^.

## Supplementary information

Supplementary Information.

## Abbreviations

ART: Artemisinin
*A. annua*: *Artemisia annua*
miRNA: MicroRNA
TF: Transcription factor
